# Inadequate Lopinavir Concentrations With Modified 8-hourly Lopinavir/Ritonavir 4:1 Dosing During Rifampicin-based Tuberculosis Treatment in Children Living With HIV

**DOI:** 10.1097/INF.0000000000004047

**Published:** 2023-07-20

**Authors:** Chishala Chabala, Anna Turkova, Monica Kapasa, Kristen LeBeau, Chimuka H. Tembo, Kevin Zimba, Lubbe Weisner, Khozya Zyambo, Louise Choo, Chalilwe Chungu, Joyce Lungu, Veronica Mulenga, Angela Crook, Diana Gibb, Helen McIlleron

**Affiliations:** 1 University of Zambia, School of Medicine, Department of Paediatrics, Lusaka, Zambia; 2 University of Cape Town, Faculty of Health Sciences, Department of Medicine, Division of Clinical Pharmacology, Cape Town, South Africa; 3 University Teaching Hospital-Children’s Hospital, Lusaka, Zambia; 4 Medical Research Council–Clinical Trials Unit at University College London, Institute of Clinical Trials and Methodology, London, United Kingdom; 5 Wellcome Centre for Infectious Diseases Research in Africa (CIDRI-Africa), Institute of Infectious Disease and Molecular Medicine, University of Cape Town, Cape Town, South Africa

**Keywords:** Lopinavir/ritonavir, rifampicin, pharmacokinetics, tuberculosis, HIV

## Abstract

**Background:**

Lopinavir/ritonavir plasma concentrations are profoundly reduced when co-administered with rifampicin. Super-boosting of lopinavir/ritonavir is limited by non-availability of single-entity ritonavir, while double-dosing of co-formulated lopinavir/ritonavir given twice-daily produces suboptimal lopinavir concentrations in young children. We evaluated whether increased daily dosing with modified 8-hourly lopinavir/ritonavir 4:1 would maintain therapeutic plasma concentrations of lopinavir in children living with HIV receiving rifampicin-based antituberculosis treatment.

**Methods:**

Children with TB/HIV coinfection weighing 3.0 to 19.9 kg, on rifampicin-based antituberculosis treatment were commenced or switched to 8-hourly liquid lopinavir/ritonavir 4:1 with increased daily dosing using weight-band dosing approach. A standard twice-daily dosing of lopinavir/ritonavir was resumed two weeks after completing antituberculosis treatment. Plasma sampling was conducted during and 4 weeks after completing antituberculosis treatment.

**Results:**

Of 20 children enrolled; 15, aged 1 to 7 years, had pharmacokinetics sampling available for analysis.Lopinavir concentrations (median [range]) on 8-hourly lopinavir/ritonavir co-administered with rifampicin (n=15; AUC_0-24_ 55.32 mg.hr/L [0.30, 398.7], C_max_3.04 mg/L [0.03, 18.6]; C_8hr_ 0.90 mg/L [0.01, 13.7]) were lower than on standard dosing without rifampicin (n=12; AUC_24_ 121.63 mg.hr/L [2.56, 487.3]; C_max_ 9.45 mg/L [0.39, 26.4]; C_12hr_ 3.03 mg/L [0.01, 17.7). During and after rifampicin co-treatment, only 7/15 (44.7%) and 8/12 (66.7%) children, respectively, achieved targeted pre-dose lopinavir concentrations ≥1mg/L.

**Conclusion:**

Modified 8-hourly dosing of lopinavir/ritonavir failed to achieve adequate lopinavir concentrations with concurrent antituberculosis treatment. The subtherapeutic lopinavir exposures on standard dosing after antituberculosis treatment are of concern and requires further evaluation.

## Introduction

Tuberculosis (TB) and HIV co-infection is common in children, particularly in TB-HIV endemic countries where options for antiretroviral treatment (ART) with standard rifampicin-based regimens are limited (1, 2). Until recently lopinavir/ritonavir (LPV/r) with two nucleoside reverse transcriptase inhibitors (NRTI) was the preferred regimen in children <3 years initiating first- or second-line ART. Currently it remains an alternative to dolutegravir-based ART and is the preferred second-line option in young children experiencing treatment failure on dolutegravir-based ART for whom child-friendly formulations of other protease inhibitors are not yet available(3).

Concomitant administration of standard doses of LPV/r in a 4:1 ratio with rifampicin is problematic. Rifampicin induces CYP3A4 and p-glycoprotein expression resulting in reductions in lopinavir pre-dose concentrations by as much as 90% when standard doses of LPV/r are used(4). Super-boosting of lopinavir with additional ritonavir in a 1:1 ratio is effective in countering the effect of rifampicin and is the preferred option for co-administration with rifampicin in children(5). However, in many low- and middle-resource settings, this ‘super-boosting’ is not feasible as suitable ritonavir formulations are not available. The alternative approach of double-dosing LPV/r, though effective in adults, achieved suboptimal concentrations in young children receiving oral LPV/r 4:1 liquid formulation(6, 7).

Although the roll-out of dolutegravir is expected to simplify TB-HIV co-treatment(8), alternative LPV/r dosing approaches are still needed for children with TB-HIV co-infection who experience adverse effects or treatment failure on dolutegravir and are unable to receive efavirenz due to young age or suspected non-nucleoside reverse transcriptase inhibitor (NNRTI) resistance.

Model-based simulations predicted that increasing the daily dose of the commercially available LPV/r 4:1 liquid formulation, together with reduction of the dosing interval from 12- to 8-hourly could maintain recommended lopinavir concentrations of 1 mg/L or above in 95% of children(9). Rabie et al. demonstrated that this approach achieved the target pre-dose concentrations of lopinavir (≥1 mg/L) in two thirds of children with no serious adverse events(10), but fell short of the model-predicted 95% target. We aimed to assess whether an increased daily dose of LPV/r, administered 8-hourly, would achieve adequate lopinavir blood concentrations in HIV-infected children receiving rifampicin-based TB treatment.

## Methods

This was a prospective pharmacokinetic study nested in the SHINE trial (ISRCTN63579542) (11). Children living with TB/HIV, weighing 3.0 to <20 kg, on LPV/r -based ART and rifampicin-containing TB treatment were enrolled in Lusaka, Zambia. Children were excluded if they had pre-existing hepatic disease or liver enzymes levels more than twice the upper limit of normal.

Children receiving LPV/r (4:1), administered as Kaletra® oral liquid (Abbvie Inc., Chicago, IL USA), were switched from 12-hourly to 8-hourly dosing strategy. Eight-hourly LPV/r was dosed according to weight-bands with children receiving 20 to 22mg/kg in the highest 18-19.9kg weight-band and 31 to 40mg/kg in the lowest 3-3.9kg weight-band. The doses of LPV/r 4.1 were adjusted 11 to 33% upwards compared to the dosages used by Rabie *et al.,* in increments pragmatic to administrator using the liquid formulation(10) (see Table, Supplemental Digital Content 1). At the time of the study, LPV/r was recommended for children <5 years old initiating ART in Zambia as preferred first-line ART(12) but super-boosting with additional ritonavir for co-treatment with rifampicin was not practiced due to non-availability of single formulated ritonavir. LPV/r was administered in combination with 2 NRTIs (abacavir or zidovudine with lamivudine). Children received rifampicin 15 (10–20) mg/kg co-formulated with isoniazid 10 (7–15) mg/kg, administered as dispersible fixed-dose combination tablets of rifampicin and isoniazid (RH) 75/50mg using WHO recommended weight bands for the continuation phase of TB treatment(13). Dosing was switched to WHO-recommended 12-hourly LPV/r two weeks after stopping rifampicin-based TB treatment.

Two intensive pharmacokinetic sampling days were conducted to assess lopinavir plasma concentrations: on 8-hourly LPV/r dosing; and 2 weeks after returning to 12-hourly dosing. The children were fasted before pharmacokinetic sampling until at least 1-2 hours after the dosing depending on the age of the patient. Samples were obtained prior to the LPV/r dose and at 1, 2, 4, 6 and 8hrs post-dose on 8-hourly dosing, with an additional 12-hour post-dose sample on 12-hourly dosing. Plasma concentrations of lopinavir and ritonavir were determined using validated liquid chromatography-mass spectrometry at the University of Cape Town pharmacology laboratory using methods previously described(10, 14). The lower limits of quantification of the lopinavir and ritonavir assays were 0.0195 mg/L and 0.00488 mg/L, respectively.

A therapeutic efficacy target of pre-dose lopinavir concentration of ≥1.0 mg/L was used as the primary pharmacokinetic endpoint. Proportions of children with C_8hr_ or C_12hr_ below this target during rifampicin co-treatment or post-rifampicin treatment, respectively, were assessed. Association between the primary endpoint with patient parameters were determined using t-test and chi-square tests. Geometric mean ratio (GMR), with 90% confidence interval (CI), for the AUC_24_, C_8hr_, C_12hr_, C_max_ and T_half_ were compared during the two time periods for children with paired observations. AUC_24_ was derived by multiplying AUC_8_ and AUC_12_ by 3 and 2 respectively. Non-compartmental analysis was used to derive the pharmacokinetic parameters using Stata version 17.0 (StataCorp, College Station, Texas, USA).

## Results

Of 20 participants enrolled, 16 underwent intensive sampling and provided 15 (174 sampling points on rifampicin) and 12 (84 sampling points without rifampicin) evaluable pharmacokinetics profiles for analysis. The pharmacokinetic profile from one child with undetectable lopinavir concentrations during rifampicin treatment was excluded from the analysis. Four participants missed the 2^nd^ sampling day (off rifampicin), 3 due to COVID-19 restrictions and one due to relocation. The median (range) age at enrollment was 3 (1 to 7) years with median (IQR) weight-for-age z-scores (WAZ) of -1.6 [-2.3, -0.9]. Five were ART-naïve while the rest were on LPV/r -based ART at enrollment with a median (IQR) duration of ART of 4.2 (2.7 to 17.4) months. All children received abacavir/lamivudine as the NRTI backbone. The median (IQR) lopinavir doses were 69.8 (68.1, 75.0) vs. 26.6 (24.1, 27.3) mg/kg per day, during 8-hourly and 12-hourly dosing, respectively. The median rifampicin (IQR) dose was 15.2(13.4, 17.4) mg/kg/day (Table 1).

**Figure 1:**
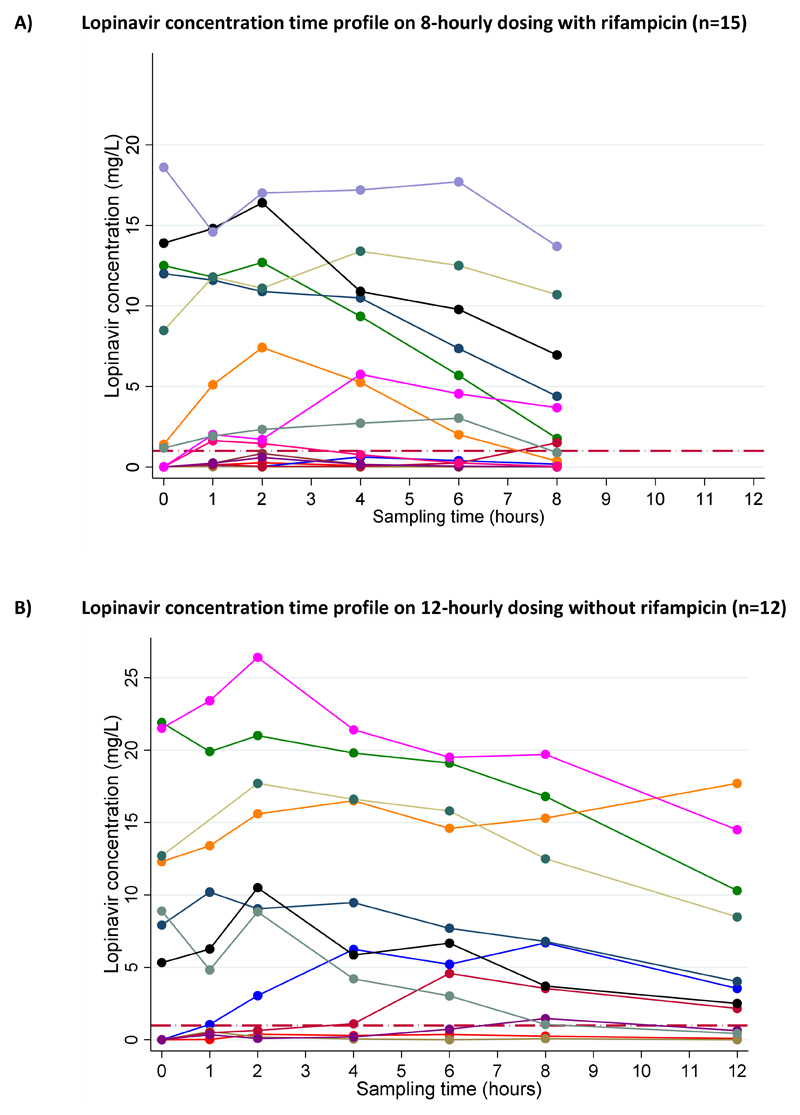
Pharmacokinetic profiles of lopinavir during co-treatment with rifampicin (A) and post-tuberculosis treatment (B). Each line in A and B represents the pharmacokinetic profile for individual participants sampled in the first and second intensive pharmacokinetic sampling session. The dotted red horizontal line represents the reference Lopinavir pre-dose target concentration of 1 mg/L.

The median (IQR) lopinavir concentrations (AUC_24_ 55.32 mg.hr/L (5.61, 222.18); C_max_ 3.04 mg/L (0.62, 12.70); C_8hr_ mg/L 0.90 (0.04, 4.39)) during treatment with rifampicin were lower than after rifampicin treatment (AUC_24_ 121.63 mg.hr/L (35.85, 353.81); C_max_ 9.45 mg/L (3.03, 17.70); C_12hr_ 3.03 mg/L (0.543, 9.39)). Only 7/15 (44.7%) achieved the recommended lopinavir pre-dose concentration of ≥1mg/L during rifampicin treatment compared to 8/12 (66.7%) without rifampicin. This result was despite higher milligram per kilogram lopinavir dose (median 23.3 mg/kg) in 8-hourly doses during treatment with rifampicin compared to the 12-hourly doses (median 13.3 mg/kg) without rifampicin (Figure 1 and Table 1). The pre-dose lopinavir concentration was 65% lower during rifampicin treatment versus without rifampicin (C_8hr_ geometric mean ratio (GMR) 0.35, 90%CI 0.30, 0.83). Similarly lower lopinavir exposures over 24h were observed during rifampicin treatment (AUC_24_ GMR 0.35, 90%CI 0.21, 0.61; C_max_ GMR 0.39, 90%CI 0.24, 0.64) (Table 1). There was no association between age(p=0.74) and weigh-for-age-z score (p= 0.13) with the pre-dose lopinavir concentrations.

Viral load (VL) measurements were performed as per national guidelines and were available for 6 participants at enrollment, 4 of whom had VL>1000copies/mL. VL results after TB treatment were available for 9 participants (average ART duration of 9.6 months); 2 participants had post-treatment VL load >1000 copies/mL while 4 and 3 participants had VL between 50-1000 copies/mL and <50 copies/mL, respectively. Overall, there was a 4-log drop in VL between enrollment and post-TB treatment.

We obtained retrospective data on drug storage and compliance to treatment; 4/10 of the caregivers reported storing the Kaletra® syrup in the refrigerator at home, 6/10 stored the drug at room temperature, and the rest provided no information on drug storage. Only 3 of the caregivers reported facing difficulties administering the drugs.

There were 3 serious adverse events recorded involving 2 participants. One participant was hospitalized on 2 separate occasions for pneumonia and acute gastroenteritis while the other was treated for a urinary tract infection. Neither required discontinuation of the intervention.

## Discussion

In our study, the modified 8-hourly approach with increased weight-band doses of liquid LPV/r 4:1 failed to achieve adequate lopinavir concentrations in children who were also receiving rifampicin. Lopinavir concentrations were low both on 8-hourly dosing during rifampicin treatment and on standard 12-hourly dosing after completing rifampicin-based TB treatment. Less than half (47%) of the children achieved the recommended concentration of 1 mg/L for lopinavir plasma trough concentrations, while on rifampicin and only two thirds (67%) achieved the target off rifampicin, while taking standard dose LPV/r. Exposures and peak concentrations were low with large inter-individual variability of the pharmacokinetic parameters observed both during and after rifampicin treatment.

Despite the higher 8-hourly doses of LPV/r in our study (21.3-31.6 mg/kg), compared to those used by Rabie *et al.* (20.3-22.4 mg/kg), lopinavir exposures were lower in our study. The approach failed in both studies with insufficient numbers of children maintaining adequate lopinavir concentrations (we report 47%, vs. Rabie’s 64%). Both studies used the 15 mg/kg (range 10-20) dose of rifampicin, currently recommended by WHO guidelines. Rabie *et al.* used LPV/r doses predicted to achieve the target lopinavir trough concentration for co-administration with rifampicin dose of 10 mg/kg (8–12) which were recommended prior to the WHO 2010 revision of pediatric anti-TB dosing. The children in our study were older than those studied by Rabie et al. (median 3.2 years vs. 1.3 years). Our study cohort also had comparatively lower median weight-for-age z-score. Poor nutritional status can be associated with low exposure and higher variability of lopinavir in infants and children because of reduced bioavailability (15, 16, 17); however, we found no association between the pharmacokinetic parameters of lopinavir and nutritional status. Importantly, in our study, lopinavir pre-dose concentrations without TB treatment were lower compared to those in other studies that achieved pre-dose concentrations ≥ 1mg/L in 87-100% of children (7, 10, 14, 18), suggesting that factors other than TB treatment contributed to the reduced exposures in our study.

The oral solution of LPV/r 4:1 is associated with reduced lopinavir exposures compared to the solid formulations (19), especially in children under age 6 months. However, our cohort did not include children under age one year, and we found no significant association between age and the lopinavir trough concentrations. Despite this, exposures without TB treatment were roughly half of those reported for children treated with LPV/r liquid formulations in other studies(7, 18, 19). It is unclear to what extent our findings of inadequate lopinavir concentrations observed can be attributed to the study population, bioavailability of the formulation used, storage conditions, adherence difficulties or other factors. Genetic polymorphisms may also contribute to high variability in lopinavir concentrations(20). Low lopinavir concentrations without concomitant rifampicin treatment were observed by Verweel *et al.* in Dutch children receiving capsule and liquid formulations, with only 70% (16/23) achieving therapeutic concentration; the lowest concentrations were observed in children younger than age 2 years in whom high lopinavir clearance is common (21).

The formulations used in the current study were supplied by the national HIV program and were dispensed within their shelf-lives. It is recommended that LPV/r should be refrigerated at 2-8° C or used within 6 weeks of dispensing if kept at room temperature (25°C) (22). Six participants reported lack of refrigeration at home and stored the drugs at room temperature in the house. We are unable to evaluate whether storage conditions impacted the formulation’s bioavailability; however, in warmer climates the stability and potency of formulations requiring cold chain may not be assured (23). Although we obtained full history of adherence on the 3 days prior to the sampling day from the caregiver and treatment was directly observed in the clinic on the sampling day, 3 caregivers retrospectively reported challenges in compliance related to the 8-hourly dosing frequency during treatment. LPV/r liquid is unpalatable making administration difficult, and this too might affect adherence to the intensified dosing regimen. (18, 23).

All the participants enrolled in the study were on the same ART regimen (LPV/r with abacavir/lamivudine), as well as co-trimoxazole and vitamin supplements, and none reported taking any other medications that could interfere with lopinavir concentrations at the time of the sampling. Whether or not the low lopinavir concentrations observed could have impacted patient outcomes is unclear as the study was not designed or powered for such evaluation. In our study two-thirds of participants (7/9) with available post-TB treatment VL test results had HIV VL below 1000 copies/mL; however, only three of nine patients had HIV VL <50 copies/mL. Low proportion of children with virological suppression could be explained by insufficient duration on ART; in our study, most (7/9) patients received ART for less than 12 months before the post TB treatment HIV VL was measured. In the SHINE trial, VL results at weeks 24 and 48, available for 90 and 82 of the 127 CLWH, revealed, respectively. 45% and 61% had VL <1000 copies/mL, respectively. Children on LPV/r-based regimens (n=43) tended to have lower rates of VL <1000 copies/mL (50% vs. 71%, p=0.056, by week 48)(24).

The study is limited by the inability to sample all the patients recruited at both pharmacokinetic sampling days reducing the power of the study given the large variability observed in lopinavir concentrations in children. Four participants were not sampled post-TB treatment and therefore did not contribute to the comparisons of pharmacokinetic parameters during vs after TB treatment.

LPV/r remains one of the most widely used protease inhibitors available for children in public health programs in low- and middle-resource settings, and in the absence of child-friendly ritonavir formulations for super-boosting, alternative dosing strategies are required for children with HIV/TB. This study highlights the importance of conducting dose-optimization studies in different pediatric populations to confirm model-predicted dosing for one of the alternative co-treatment strategies. This study and other pediatric PK studies (7, 10) demonstrated that increasing the daily dose and frequency to 8-hourly of standard LPV/r 4:1 liquid formulation resulted in suboptimal therapeutic lopinavir levels. Although the interpretation of our findings is complicated by lower-than-expected lopinavir exposures without rifampicin, this study supports the findings of Rabie et al.(10), which used lower model-predicted 8-hourly doses, and in which the approach failed to counter the inducing effect of rifampicin. The 8-hourly dosing approach cannot therefore be relied upon as an alternative option for TB co-treatment. This supports the rapid roll out of dolutegravir-based treatment in TB endemic countries where the challenges of HIV/TB co-treatment persist, as well as the roll out of child-friendly ritonavir formulations for lopinavir superboosting for children unable to take dolutegravir. The sub-therapeutic concentrations of lopinavir with the use of LPV/r liquid without rifampicin co-administration requires further evaluation and supports the use of more heat stable formulations such as granules or tablets especially in environments where storage conditions may not be assured.

## Supplementary Material

Supplemental Digital Content (Including Separate Legend)

## Figures and Tables

**Table 1:** Patient Characteristics and Pharmacokinetic Measures During 8-hourly and 12-hourly Lopinavir/Ritonavir Dosing

Patient characteristics			
	LPV/r 8-hourly during TB treatment	LPV/r 12-hourly after TB treatment	p-value
Number of patients	15	12	
Sex, male	10	7	0.66
Weight on PK* sampling, median (IQR) mg	12.1(11.2, 14.8)	13.5(12.0, 14.7)	0.61
WAZ, median (IQR)	-1.2 (-1.6, -0.5)	-1.0 (-2.2, -0.7)	0.53
WHZ, median (IQR)	0.2 (-0.7, 0.9)	0.0 (-0.2, 1.3)	0.79
Lopinavir dose, median (IQR) mg/kg/day	69.8 (68.1,75.0)	26.6 (24.1, 27.3)	<0.01
**Pharmacokinetic characteristics**			
	n=15	n=12	GMR*** (90% CI)
AUC 24**, median (IQR)mg.hr/L	55.32 (5.61, 222.18)	121.63 (35.85, 353.81)	0.35 (0.21, 0.61)
C_max_, median (IQR) mg/L	3.04 (0.62, 12.70)	9.45 (3.03, 17.70)	0.39 (0.24, 0.64)
C_min_, median (IQR) mg/L	0.90 (0.04, 4.39)	3.03 (0.543, 9.39)	0.35 (0.30, 0.83)
t-half, median (IQR) hr	2.5(1.33, 6.18)	6.56 (4.5, 9.27)	0.55 (0.30, 1.01)
C_min_ ≥1mg/L, n (%)	7 (46.7)	8 (66.7)	p=0.44

* PK; pharmacokinetics

** AUC_24_ was derived by multiplying 3 x AUC_8_ during rifampicin co-treatment and 2 x AUC_12_ post rifampicin co-treatment

*** GMR; Geometric Mean Ratio for paired data in the 1^st^ and 2^nd^ pharmacokinetic sampling session
