# Supplementary material for: Inadequate Lopinavir Concentrations With Modified 8-hourly Lopinavir/Ritonavir 4:1 Dosing During Rifampicin-based Tuberculosis Treatment in Children Living With HIV
Source: Pediatr Infect Dis J. Author manuscript; Available in PMC 2023 Sep 16. (PMC10501348; doi:10.1097/INF.0000000000004047)
Supplement: Supplemental Digital Content (Including Separate Legend) [file EMS178635-supplement-Supplemental_Digital_Content__Including_Separate_Legend_.docx]

**Supplemental Digital Content 1.** Weight band doses used in the SHINE Lopinavir study compared to the DATic study and WHO standard dosing recommendation.

|  | **SHINE 8 hourly doses (ISRCTN63579542)** | | | | **DATic 8 hourly doses (NCT01637558)** | | | | **Standard 12 hourly WHO doses** | | | |  |  |
| --- | --- | --- | --- | --- | --- | --- | --- | --- | --- | --- | --- | --- | --- | --- |
| **weight band** | LPV dose (mg) | mg/kg (min) | mg/kg (max) | LPV dose (ml) | LPV dose (mg) | mg/kg (min) | mg/kg (max) | LPV dose (ml) | LPV dose (mg) | mg/kg (min) | mg/kg (max) | LPV dose (ml) | % Increase in dose per day used in SHINE vs DATic doses  during TB treatment | % Increase in dose per day used in SHINE vs. standard WHO standard doses |
| 3.0 - 3.9 | 120 | 31 | 40 | **1.5** | 100 | 25.6 | 33.3 | **1.25** | 80 | 21 | 27 | **1** | 20 | 125 |
| 4.0 - 4.9 | 160 | 33 | 40 | **2** | 120 | 24.5 | 30.0 | **1.5** | 120 | 24 | 30 | **1.5** | 33 | 100 |
| 5.0 - 5.9 | 160 | 27 | 32 | **2** | 140 | 23.7 | 28.0 | **1.75** | 120 | 20 | 24 | **1.5** | 14 | 100 |
| 6.0 - 6.9 | 160 | 23 | 27 | **2** | 140 | 20.3 | 23.3 | **1.75** | 120 | 17 | 20 | **1.5** | 14 | 100 |
| 7.0 - 7.9 | 200 | 25 | 29 | **2.5** | 160 | 20.3 | 22.9 | **2** | 120 | 15 | 17 | **1.5** | 25 | 150 |
| 8.0 – 8.9 | 200 | 22 | 25 | **2.5** | 180 | 20.2 | 22.5 | **2.25** | 120 | 13 | 15 | **1.5** | 11 | 150 |
| 9.0 - 9.9 | 240 | 24 | 27 | **3** | 200 | 20.2 | 22.2 | **2.5** | 120 | 12 | 13 | **1.5** | 20 | 200 |
| 10.0 - 11.9 | 280 | 24 | 28 | **3.5** | 240 | 20.2 | 24.0 | **3** | 160 | 13 | 16 | **2** | 17 | 163 |
| 12.0 - 13.9 | 320 | 23 | 27 | **4** | 280 | 20.1 | 23.3 | **3.5** | 160 | 12 | 13 | **2** | 14 | 200 |
| 14.0 -15.9 | 320 | 20 | 23 | **4** | 280 | 17.6 | 20.0 | **3.5** | 200 | 13 | 14 | **2.5** | 14 | 140 |
| 16.0 - 17.9 | 360 | 20 | 23 | **4.5** | 320 | 17.9 | 20.0 | **4** | 200 | 11 | 13 | **2.5** | 13 | 170 |
| 18-19.9 | 400 | 20 | 22 | **5** | 360 | 18.1 | 20.0 | **4.5** | 200 | 10 | 11 | **2.5** | 11 | 200 |
